# Genetic Background and Clinical Characters of Pediatric Chronic Pancreatitis: Data and Implications from the East

**DOI:** 10.1155/2017/7548753

**Published:** 2017-02-28

**Authors:** Muyun Liu, Tian Xia, Di Zhang, Lianghao Hu, Zhuan Liao, Chang Sun, Zhaoshen Li

**Affiliations:** Department of Gastroenterology, Changhai Hospital, Second Military Medical University, Shanghai, China

## Abstract

*Background.* The clinical pattern and genetic background of juvenile idiopathic chronic pancreatitis (ICP) are yet unclear. *Methods*. A retrospective study of 73 Chinese juvenile ICP patients was performed, and genetic tests were carried out to detect relevant mutations using direct sequencing technique and high-resolution melting technique. Subjects without pancreatitis served as controls. *Results.* The SPINK1 c.194+2T>C variant was present in 56.16% and 42.00% of juvenile and adult ICP patients, respectively (*p* = 0.020), but was not present in any of the control subjects. Thirty-four (46.58%) of the 73 juvenile ICP patients were male, and a significantly higher ratio of male patients in the adult group was identified (46.58% versus 64.00%, *p* = 0.022). Although most of the juvenile patients presented with abdominal pain (70/73, 95.89%), the patterns of pain attack are significantly different in patients with or without SPINK1 c.194+2T>C mutation. Patients carrying the mutation are more likely to present with recurrent acute pancreatitis (70.70%). *Conclusions.* The main symptom of pediatric ICP was abdominal pain. SPINK1 c.194+2T>C mutation had a higher occurrence in juvenile ICP patients than in adult group and typically presented with recurrent acute pancreatitis. There may be unidentified factors that lead to a greater incidence rate of ICP in adult male population.

## 1. Introduction

Chronic pancreatitis (CP) is defined as a progressive fibro-inflammatory disease characterized by the irreversible damage of the pancreas, associated with chronic or recurrent abdominal pain and impairment of exocrine and endocrine function, occasionally associated with pancreatic lump, jaundice, and ascites [1–3]. Genetic variations have been recognized as a major etiology factor for this disease [4, 5]. CP is a rare disorder in children and adolescents, which will result in excessive number of missed school days and reduction in activities, even addiction to pain medication. Frequent visit to the hospital also causes psychological impairment and self-abasement in underage patients. Researches in the last decade have proven an increasing incidence of CP in children [6].

Environmental factors such as alcoholic consumption, cigarette addiction, and diet immoderation play a smaller role in the onset of chronic pancreatitis than found in adults; thus, the etiology of juvenile CP can be distinct from adult patients [7]. The most common etiological factors of juvenile CP were idiopathic, anatomical anomalies, hyperlipidemia, and trauma. In Chinese population, 73.8% among all pediatric CP patients are idiopathic chronic pancreatitis (ICP) [6, 8] and the main symptom is multiple episodes of mild to moderate abdominal pain [9].

Childhood-onset chronic pancreatitis has distinct features in etiologies, diagnosis, and treatment methods from adult population [10, 11]. Recently, an international multicenter INSPPIRE (International Study Group of Pediatric Pancreatitis: In Search for a Cure) consortium has taken a great step forward to reveal the epidemiology, clinical characteristics, and genetic background of pediatric CP in western population [6, 12]. By investigating 146 pediatric patients from U.S., Canada, and Australia who were diagnosed with CP, they draw the conclusion that relevant pathogenic gene mutations were found in 73% of pediatric CP patients which confirmed the role of genetic variation in adolescent CP patients on the basis of a vast western population. Notably, this study reported a tight connection between serine protease inhibitor Kazal type 1 (SPINK1, OMIM 167790) c.101A>G mutation and the onset of pediatric CP.

Protease serine 1 (PRSS1, OMIM 276000), cystic fibrosis transmembrane conductance regulator (CFTR, OMIM 602421), chymotrypsin C (CTRC, OMIM 601405), and SPINK1 exonic mutation are the most frequently detected gene mutations in Caucasian population [13]. Among more than 100 reported SPINK1 variants, the SPINK1 c.101A>G mutation is most frequently detected in Caucasian population while SPINK1 c.194+2T>C has often been reported in Asian populations. Among the limited investigation concerning SPINK1 c.194+2T>C mutation in western populations, Rosendahl et al. found 2.1% CP patients from Germany who carried SPINK1 c.194+2T>C mutation in comparison to 16.2% patients who carried c.101A>G mutation [14]. However, the situation is completely different in Asian countries. SPINK1 c.101A>G mutation is less frequently found in Asian pediatric CP patients while c.194+2T>C was found in 9.37% to 31.25% of patients according to studies from Japan and Korea [15, 16]. Studies among Chinese population reveal that SPINK1 c.194+2T>C mutation is most frequently seen in Chinese CP patients [17, 18].

In order to further elucidate the diversity in genetic background and clinical characters between Asian and Caucasian pediatric CP patients as well as to address whether the genetic heterogeneity results in different clinical patterns, we performed this retrospective analysis. In this study, we only included pediatric ICP (idiopathic chronic pancreatitis) patients ruling out those with a specific pathogenesis such as pancreatic injury and anatomic abnormality to avoid complication.

## 2. Materials and Methods

### 2.1. Subjects

This was a retrospective study, and all consecutive patients with ICP that received treatment from July 2011 to March 2016 at the Department of Gastroenterology at Changhai Hospital of the Second Military Medical University were included in the study. Our endoscopic center introduced the first extracorporeal shock wave lithotripsy machine targeting pancreatic stones and is well known as the largest ERCP training center in China. So pediatric CP patients are also hospitalized in our department.

The diagnosis of CP was made in the appropriate clinical setting if there was evidence of pancreatic duct dilatation and irregularity and/or pancreatic calcification found in imaging studies [2]. Idiopathic chronic pancreatitis was diagnosed if preexisting disorders likely to cause chronic pancreatitis (hypertriglyceridemia, primary hyperparathyroidism, abdominal trauma, and pancreatic duct stenosis caused by operation), hereditary chronic pancreatitis (HCP) (as determined by family history), and excessive alcohol consumption were absolutely ruled out [19]. Juvenile idiopathic chronic pancreatitis was diagnosed with the age at the first onset of symptoms being 18.0 years or younger [9].

**Figure 1:**
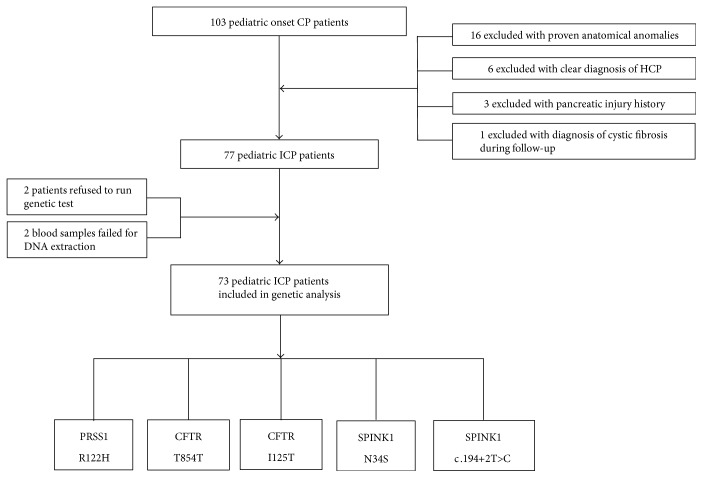
Of all the 103 pediatric ICP patients, a total of 26 patients were excluded for clear pathogenic factors such as anatomic abnormalities and pancreatic injuries.

Among all the CP patients, a total of 103 had disease onset age younger than eighteen. Sixteen of them had anatomical anomalies in pancreas and/or pancreatic duct that can be proved by computed tomography (CT) and/or magnetic resonance imaging (MRI). Family history was recorded and six pediatric patients were excluded with a clear diagnosis of HCP. Three patients were excluded with a pancreatic injury history. Notably, one patient was finally diagnosed with cyst fibrosis thus excluded from this study. A total of 77 pediatric onset CP patients were included. All 77 families were informed about the peripheral blood and genetic analysis. Two of the families refused to participate in further analysis. Among the 77 peripheral blood samples we collected, two samples degraded and failed for PCR analysis due to inappropriate storage method (Figure 1).

To compare clinical characters and genetic backgrounds of pediatric ICP patients and adult ICP patients, we randomly took 100 adult ICP patients from our database with complete chronic pancreatitis-relevant genetic background information.

The controls were healthy individuals from blood donors. “Healthy” was defined as the absence of any type of infection or known medical condition at the time of the study. We obtained 5 ml of peripheral blood from every healthy control and patient, and the samples were stored at −80°C after anticoagulation treatment with EDTA.

For deidentification, all of the samples were coded with a number to protect the privacy of the individuals during the study. All of the participants gave written informed consent to participate in this study, and the samples were processed under the approval of the Changhai Hospital Ethics Committee.

### 2.2. Genomic Analysis

As previously described, Qiagen DNeasy blood and tissue kit was used to extract genomic DNA from the blood sample. Direct sequencing was used to analyze mutations of SPINK1 gene (OMIM 167790) exon 3 and intron from polymerase chain reaction (PCR) products [18]. In brief, a primer pair was designed to amplify regions of the SPINK1 exon 3 and intron. PCR products were analyzed using direct sequencing (ABI Prism Automated DNA Sequencer model 3100, PE Applied Biosystems, USA). Analysis of exons 2 and 3 of PRSS1 gene (OMIM 602421) mutations was performed by direct sequencing of PCR products. Primer pairs were designed to detect PRSS1 gene exon 2 and exon 3.

A HRM (high-resolution melting) technique provided by the Shanghai Healo-Tech Corp was used to scan mutations of the CFTR (cystic fibrosis transmembrane conductance regulator, MIM 602421) gene as previously described [18]. All sequences were compared to the reference sequences available in GenBank of the National Center for Biotechnology Information database (https://www.ncbi.nlm.nih.gov).

### 2.3. Clinical Characters

Disease onset age was recorded according to the occurrence of disease related symptoms including acute pancreatitis, recurrent abdominal pain, chronic pancreatic pain, or radiological findings. Disease related pain patterns were defined as recurrent acute pancreatitis (RAP), recurrent abdominal pain (RP) without significant increase in serum amylase, recurrent acute pancreatitis or abdominal pain (RAP/P) without significant increase in serum amylase, chronic pancreatic pain (CPP), or no pain attack. Pancreatic calcifications include both pancreatic stones located in the main or branch pancreatic ducts and calcifications found in pancreatic parenchyma. Diabetes was diagnosed with fasting glucose level over 7.0 mmol per liter and glycated hemoglobin level over 6.5% [20]. Clinical patterns and complications were recorded when patients were first included in this study.

### 2.4. Statistical Analysis

The data are presented as means and SDs. The unpaired *t*-test for comparing quantitative data and the *χ*^2^ test for qualitative data were used when appropriate. A *p* value of 0.05 was used as the cutoff for statistical significance. Graphpad Prism (version 7) was used for the statistical analyses.

## 3. Results

### 3.1. General Information

A total of 73 pediatric chronic pancreatitis patients were included in the final analysis as shown in Figure 1. General information of the included pediatric ICP patients was analyzed. Of the 73 juvenile ICP patients, 34 (46.58%) were male (Table 1). In comparison with the sex distribution of juvenile and adult ICP patients (*n* = 100, data from published paper) among which 64% patients were male, there were significantly more male patients in adult population (*p* = 0.022). Body mass index was 19.55 ± 2.36 in pediatric ICP patients at the time of enrollment (Table 1).

### 3.2. Clinical Features

Nearly all of the 70 (95.89%) juvenile ICP patients presented with abdominal pain. Only 1 out of 73 (1.37%) pediatric ICP patients presented with jaundice. Imaging methods including CT, magnetic resonance imaging (MRI), B-ultrasound, and X-ray suggested pancreatic calcification in 58 (79.54%) pediatric ICP patients. Notably, one of these patients had negative pancreatic stone which has been proven by pancreatic duct obstruction presented in magnetic resonance cholangiopancreatography (MRCP) images and proven in endoscopic retrograde cholangiopancreatography (ERCP) (Table 1).

### 3.3. Complications

Pseudocyst was the most common complication and was noted in 9.59% (7/73) juvenile ICP patients. Ascites or pleural effusion was also relatively common in juvenile ICP patients as 2 patients (2.74%) presented with these symptoms. Both patients were hospitalized during episode of acute pancreatitis (Table 1). Diabetes on presentation was noted in 4 out of the 73 (5.48%) juvenile ICP patients at the time of enrollment. So far, none of the patients with diabetes had microvascular or macrovascular complications on presentation. One out of the four patients relied on insulin treatment. During our follow-up, another patient had endocrine function deterioration and finally started insulin treatment to steadily control blood glucose. None of the 73 pediatric ICP patients had steatorrhea at enrollment or during follow-up. Continuous supplement of pancreatic enzymes for some of the patient may have interfered with the observation of exocrine function deterioration.

### 3.4. Genetic Profile

Among the 73 pediatric ICP patients, 14 (19.18%) carried PRSS1 C.365G>A gene mutation compared to 12 (12%) in adult ICP patients; all of these patients carried PRSS1 C.365G>A heterozygous mutation. No significant difference was observed between adult and pediatric populations. Heterozygous CFTR C.2562T>G mutation was observed in 2.74% (2/73) pediatric patients compared to 2.00% (2/100) in adult patients, and no statistical difference was noticed.

Neither juvenile nor adult ICP patients had SPINK1 C.101A>G mutation. Of the 73 juvenile ICP patients, 41 (56.16%) patients had c.194+2T>C mutation as compared with 42 (42.00%) of 100 adult patients suggesting a higher mutation rate in the juvenile group (*p* = 0.002) (Table 2). Among the 41 pediatric chronic pancreatitis patients, 8 carried homozygous SPINK1 c.194+2T>C mutation and 33 carried heterozygous c.194+2T>C mutation. In addition, none of the healthy controls was found to carry any relative mutations; thus, significant differences were detected between juvenile ICP patients and healthy controls as well as between adult ICP patients and healthy controls (*p* < 0.001) (Table 2).

### 3.5. Comparison of Pediatric CP Patients with or without SPINK1 c.194+2T>C Mutations

To address the clinical features and translational implications of SPINK1 c.194+2T>C mutation, we compared the two groups of pediatric ICP patients with or without SPINK1 c.194+2T>C mutation. No difference in CP onset age or gender distribution was noticed (*p* = 0.674; *p* = 0.170, resp.). However, the onset pattern was distinctively different among these two groups of patients. Among all 41 pediatric CP patients carrying the SPINK1 intronic mutation, 29 (70.7%) presented with RAP. The rest of pediatric patients who were carrying SPINK1 c.194+2T>C mutation presented with RP (6, 14.6%), RAP/P (1, 2.4%), CPP (3, 7.3%), and no pain attack (2, 4.9%). On the other hand, among the 32 patients without SPINK1 c.194+2T>C mutation, 10 (31.3%) presented with RAP, 2 (6.3%) presented with RP, 11 (34.4%) presented with RAP/P, 8 (25.0%) presented with CPP, and 1 (3.1%) did not present with abdominal pain. Significant difference between groups was confirmed by *χ*^2^ test (*p* < 0.001) (Table 3).

Notably, all of the four patients with diabetes carried SPINK1 c.194+2T>C. But no statistical significance was shown due to the limited diabetes onset chances in children and adolescent patients.

## 4. Discussion

In the current study, we draw a picture of the clinical features and genetic backgrounds of pediatric ICP in China. In addition, we made comparison between adult and pediatric ICP patients to dig into the heterogeneity of ICP in these two patient populations. Results in our study showed that the sex distribution of juvenile and adult patients differed significantly. The percentage of male patients was higher than that in pediatric patients. According to a multicenter study initiated by the Chinese Chronic Pancreatitis Study Group to determine the nature and magnitude of CP in China, 64.99% among the 2008 patients included were male and 35.01% were female [3]. Notably, this study included chronic patients of all different etiologies. Data from Japan and western countries demonstrated elevated ICP onset rate in male population [21, 22]. Obvious difference of sex distribution between CP patients in these age groups suggested that male population is more likely to be exposed to environmental factors such as smoking, unhealthy lifestyle, and a higher BMI as well as some unknown risk factors. According to previous studies, alcohol consumption at lower levels (<50 g/day) was suggested to play a modifying role in disease development and these patients were believed to represent a subset of late-onset idiopathic CP [23]. Also, previous researches confirmed the association between smoking and CP and demonstrated that this effect is dose dependent [24–27].

In our present study, we found that almost all pediatric ICP patients had abdominal pain (95.89%). Patients with early-onset idiopathic and alcoholic chronic pancreatitis are more likely to have clinical symptoms mostly presented with abdominal pain when compared with subjects with late-onset idiopathic CP [28]. Furthermore, the presence of pain in juvenile CP was 100% according to the research data in another study [2]. On the other hand, only 1.37% patients presented with jaundice and 9.59% patients presented with pancreatic pseudocyst. On this basis, the disease related pain as well as acute pancreatitis onset should be listed as the primary factor that we need to consider in clinical treatment plans.

Genetic mutations related to pancreatitis have been studied in a wide range of populations all over the world [4]. The functions of typical mutations in genes such as PRSS1 and CFTR have been elucidated, and their prevalence has been well addressed in Caucasian populations [29–31]. Gomez-Lira et al. found that the c.194+2T>C intronic alteration could abolish SPINK1 expression at the mRNA level [32]. Studies in Chinese population these years revealed that unlike in Caucasian populations, SPINK1 c.194+2T>C was the most common mutation of SPINK1 gene in Chinese CP patients [17, 18]. This has also been proven in our present study as both juvenile and adult ICP patients were detected with a SPINK1 c.194+2T>C mutation while none of the healthy controls presented with any mutation of SPINK1. In addition, we found that the percentage of the patients carrying SPINK1 c.194+2T>C mutation was even higher in pediatric ICP patients than in adult ICP patients. This result indicates that genetic factors play a more important role in childhood ICP rather than that in adult ICP, suggesting other factors such as environment, diet, sexuality, and lifestyle may impact adult ICP patients to a greater extent with time effect. Furthermore, as c.194+2T>C mutation was not seen in any of the healthy controls, there lies a possibility that the test for c.194+2T>C mutation can serve as diagnostic criteria or even as an antenatal diagnosis method. Notably, genetic analysis of the 73 pediatric ICP patients detected PRSS1 C.365G>A mutation. According to previous studies, PRSS1 gene mutations are considered a typical feature for hereditary chronic pancreatitis [33]. However, as far as we are concerned, SPINK1 mutations have also been recognized as closely related to familial pancreatitis [33, 34]. So we analyzed the characters of all the 73 pediatric ICP patients.

The high frequency of SPINK1 c.194+2T>C mutation inspired us to dig into its clinical implications. Surprisingly, we found that the pain pattern at disease onset is distinctly different in pediatric ICP patients with or without SPINK1 c.194+2T>C mutation. The most common type of pain attack in pediatric patients carrying the intronic mutation is recurrent acute pancreatitis which is found in 70.7% of all cases. However, the pattern of pain attack in patients without SPINK1 c.194+2T>C mutation can be characterized as RAP, RP, RAP/P, and CPP in a decentralized fashion. This result gives us a hint that, for pediatric patients suffering from RAP attack, an examination of related gene mutation should be recommended. Whether this finding can serve as an implication of the genetic connection between RAP and CP is quite fascinating. Our group has initiated a screening of genetic mutations in Chinese recurrent pancreatitis patients and hopefully this study could further reveal the whole picture.

Due to the limited time of disease progression, very few pediatric ICP patients presented with endocrine and exocrine function impairment. However, all of the 4 patients with diabetes mellitus carried SPINK1 c.194+2T>C mutation. In the previous studies from our group, we found that SPINK1 c.194+2T>C mutation is a predisposing factor for the earlier onset of pancreatic diabetes [18] which is consistent with this result.

According to a recent publication from Korea, pancreatic duct stones occurred more frequently in patients with the c.194+2T>C pathogenic variant [35]. It gives us a hint to look into this situation from another clinical prospective. As a majority of chronic pancreatitis patients came to our treatment center hoping to undergo interventional treatments including ESWL and ERCP to resolve pancreatic ductal obstruction, the occurrence of pancreatic stone might be higher. We are undergoing further analysis to elucidate this discovery.

According to the 2012 American Community Survey, Asian American comprises 15.5 million out of the whole population in the United States. Moreover, Asian population has been growing in North America, Austria, and New Zealand. Thus, gene diversity should be considered for a better coverage of different races. As the frequency of SPINK1 c.194+2T>C is rather in Chinese chronic patients and distinct clinical features have been addressed, we propose that this intronic variant should arouse more attention in researchers and clinicians all over the world to better facilitate the practice of precision medicine.

## 5. Conclusion

By comparing different groups of ICP patients, we concluded that SPINK1 c.194+2T>C mutation had a higher occurrence in juvenile ICP patients than in the adult group. There are more male patients in adult ICP population than in pediatric patients. More patients carrying SPINK1 c.194+2T>C presented with recurrent acute pancreatitis at disease onset. We should pay more attention to pathogenic intronic variants in relevant studies among different races and generations.

## Figures and Tables

**Table 1:** Clinical characters of juvenile ICP patients.

	Children and adolescent CP patients (*n* = 73)
Age at onset	10.37 ± 3.42
Male	34 (46.58%)
Body mass index (Kg/m^2^)	19.55 ± 2.36
Pain	70 (95.89%)
Jaundice	1 (1.37%)
Lump	0 (0.00%)
Pancreatic calcification	58 (79.45%)
*Complications*	
Ascites and/or pleural effusion	2 (2.74%)
Portal hypertension	0 (0.00%)
Pseudocyst	7 (9.59%)
Diabetes mellitus	4 (5.48%)
Steatorrhea	0 (0.00%)

ICP, idiopathic chronic pancreatitis; CP, chronic pancreatitis.

**Table 2:** Genetic characteristics of patients with idiopathic chronic pancreatitis and controls.

	Pediatric ICP patients (*n* = 73)	Adult ICP patients (%) (*n* = 100)	Healthy controls (%) (*n* = 100)
PRSS1 c.365G>A	14/73 (19.18%^#^)	12/100 (12.00%)	0 (0%)
CFTR c.2562T>G	2/73 (2.74%)	2/100 (2.00%)	0 (0%)
SPINK1 c.101A>G	0/73 (0%)	0/100 (0.00%)	0 (0%)
c.194+2T>C^∗^	41/73 (56.16%)	42/100 (42.00%)	0 (0%)

ICP: idiopathic chronic pancreatitis; PRSS1, protease serine 1; CFTR, cystic fibrosis transmembrane conductance regulator; SPINK1: serine protease inhibitor Kazal type 1; ^#^the percentage refers to the proportion of patients within each group. ^∗^*p* = 0.02 juvenile ICP versus adult ICP.

**Table 3:** Comparison of juvenile ICP patients with or without the SPINK1 c.194+2T>C mutations.

	Juvenile patients carrying the mutation (*n* = 41)	Juvenile patients without the mutation (*n* = 32)	*p* value
Age at onset (years, mean ± SD)	10.22 ± 2.91	10.56 ± 4.02	0.674
Female	19 (46.3%)	20 (65.2)	0.170
*Clinical features*			
Type of pain attack			<0.001
RAP	29 (70.7%)	10 (31.3%)	
RP	6 (14.6%)	2 (6.3%)	
RAP/P	1 (2.4%)	11 (34.4%)	
CPP	3 (7.3%)	8 (25.0%)	
No pain attack	2 (4.9%)	1 (3.1%)	
Jaundice	1 (2.4%)	0 (0.0%)	0.307
Pancreatic calcification	32 (78.0%)	26 (81.3%)	0.737
*Complications*			
Pseudocyst	4 (9.8%)	3 (9.4%)	0.956
Diabetes mellitus	4 (9.8%)	0 (0%)	0.069
Steatorrhea	0 (0.00%)	0 (0.00%)	na

SPINK1, serine protease inhibitor Kazal type 1; SD, standard deviation; RAP, recurrent acute pancreatitis; RP, recurrent abdominal pain; RAP/P, recurrent acute pancreatitis or abdominal pain without significant increasing in serum amylase; CPP, chronic pancreatic pain.

## Abbreviations

ICP: Idiopathic chronic pancreatitis
SPINK1: Serine protease inhibitor Kazal type 1.
